# Better than Membranes at the Origin of Life?

**DOI:** 10.3390/life7020028

**Published:** 2017-06-20

**Authors:** Helen Greenwood Hansma

**Affiliations:** Department of Physics, University of California, Santa Barbara, CA 93106, USA; hhansma@physics.ucsb.edu or helen.hansma@gmail.com; Tel.: +1-805-729-2119

**Keywords:** membraneless organelles, membrane-less organelles, origin of life, origins of life, ribosomes, RNPs, ribonucleoprotein particles, Muscovite mica, mechanical energy

## Abstract

Organelles without membranes are found in all types of cells and typically contain RNA and protein. RNA and protein are the constituents of ribosomes, one of the most ancient cellular structures. It is reasonable to propose that organelles without membranes preceded protocells and other membrane-bound structures at the origins of life. Such membraneless organelles would be well sheltered in the spaces between mica sheets, which have many advantages as a site for the origins of life.

## 1. Introduction

Membranes are fragile. They leak, acquire and lose molecules, swell, and rupture. The plasma membranes of free-living cells are protected by cell walls, in Bacteria and Archaea; and thick protein coats protect single-celled Eukaryotes such as *Paramecium* [1].

Membranes around living cells come in two basic types. Archaea have membranes where the lipid bilayer is formed from isoprenoid ether-linked lipids. Bacteria and Eukaryotes have membranes with lipid bilayers containing fatty acid esters such as those found in triglycerides [2,3]. This is a problem for ‘membranes first’ [4] theories of the origins of life. If there were membranes before the cellular contents within the membranes were alive, how did there come to be two types of membranes surrounding cells whose contents do not correspond to these two types of membranes? Both types of membrane lipid contain 3-carbon glycerol backbones, and the two alkyl chains are on adjacent carbons of glycerol.

There is an alternative to membranes at the origins of life. Organelles without membranes are found in all types of organisms: multi-cellular organisms, whose cells have nuclei (Eukaryotes), and single-celled organisms without nuclei (Bacteria and Archaea) [5].

## 2. Location of Membraneless Organelles at the Origins of Life?

**Figure 1:**
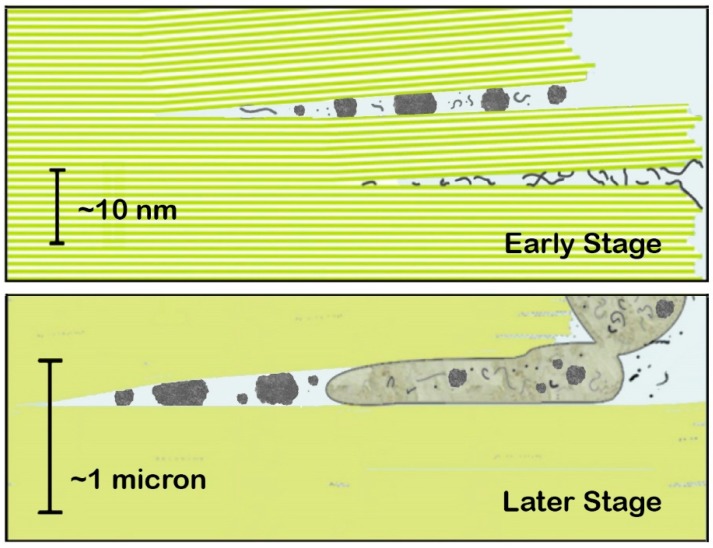
Diagrams of an origin of life between mica sheets under water with membraneless organelles and other prebiotic structures. Green lines and areas represent green Muscovite mica. Potassium (K) ions in the spaces between sheets (white lines in the Early Stage) hold sheets together. The various gray structures represent extended polymers (linear structures), molecular aggregates and membraneless organelles (gray globules), and protocells (large budding structure in the Later Stage). Protocells have a ‘protoplasm’ that is not only water. 10-nm scale bar in the Early Stage is the thickness of 10 mica sheets. 1-micron scale bar in the Later Stage is the thickness of 1000 mica sheets. Mechanical energy of moving mica sheets causes processes such as the blebbing off of protocells. Membraneless organelles and molecular aggregates in the Early Stage are tiny (~1–5 nm diameter). The small and medium-sized globules in the Later Stage are ~66 nm and ~130 nm, smaller than ribosomes, which are ~200–300 nm diameter. Nucleoli in present-day cells are ~1–10 microns in diameter, larger than the protocells in the Later Stage. Adapted from [6].

For both membrane-bound and membrane-less organelles, the spaces between mica sheets provide shelter and many other advantages as a site for the origins of life (Figure 1) [6,7,8]. Mica sheets are bridged by potassium (K) ions, found at high concentrations in all living cells but generally absent from hypotheses for the origins of life. Mica’s crystal lattice resembles clay lattices, which have been used as a support and/or a catalyst for research on the synthesis of prebiotic polymers [9]. Both mica and clay lattices have a hexagonal grid of anions on their surfaces, with a periodicity of 0.5 nm. This is also the periodicity of extended single-stranded nucleic acids and carbohydrate polymers. Clays swell and shrink during wetting and drying, unlike mica, which only gains and loses water between some of its sheets in a more gentle process. See Figure 6 in [6] for images of water drying between mica sheets. The thickness of the water layer between mica sheets varies from sub-nanometer to microns or more. Like clays and other porous rocks, mica provides protection and partial isolation from the bulk prebiotic environment.

**Figure 2:**
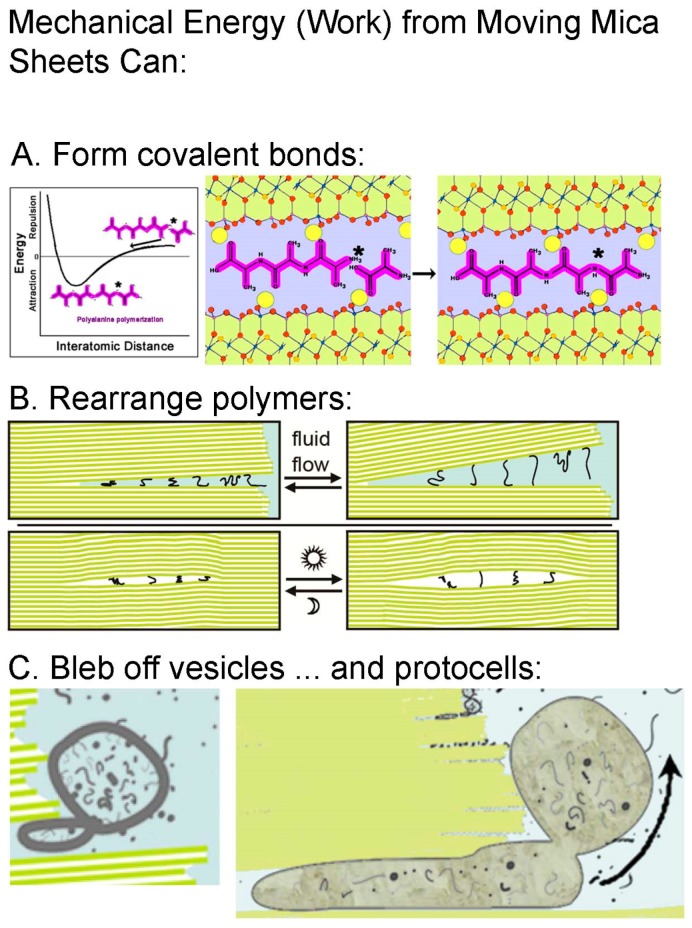
Work done by moving mica sheets is capable of mechanochemistry. (**A**) On a picometer scale, mechanochemistry can form covalent bonds, even by simply grinding reactants together [17] The diagrams show moving mica sheets mechanochemically causing the polymerization of alanine by pushing molecules of alanine and tri-alanine into the attractive regime of the potential energy well. Yellow circles are K-ions, spaced 1 nm apart on the mica surfaces. (**B**) On a nanometer scale, mechanical energy can stretch and rearrange polymeric molecules. (**C**) On a micron scale, mechanical energy can cause division of vesicles and protocells. See Figure 1 caption for descriptions of the mica diagrams in (**B**,**C**), and ref. [6] for more detail.

Mica provides another big advantage over other porous rocks: mechanical energy (Figure 2) [6,10]. The formation of chemical bonds occurs by bringing atoms or molecules into close proximity, where there is an attractive force between the atoms and/or molecules. See Figure 2A for a diagram of this process, showing how mechanochemistry might cause a reaction between the amino acid alanine and the peptide tri-alanine to form the peptide tetra-alanine. Mechanical energy is capable of forming chemical bonds. The mechanical energy of moving mica sheets comes from water movements or temperature changes. Both water movements and temperature changes cause mica sheets to move apart and together. Energy is vital for the transition from non-living to living, and mechanical energy from moving mica sheets would be an endless energy source for the origins of life, because water movements and temperature changes occur without stopping. Mechanical energy and forces are ubiquitous in living cells at all size scales [11,12,13], perhaps because forces and mechanical energy were involved in the transition from non-living to living matter.

Mechanical energy is the product of the forces acting on the mica sheets and the distances of the open-and-shut motions of the mica sheets. Moving mica sheets can generate a wide range of energies. The mechanical energy from moving mica sheets depends on the spring constant of the mica spring, which is proportional to the thickness of the mica spring. The thickness of the mica spring varies by 1-nm increments, because single mica sheets are 1 nm thick.

Mechanical energy may have preceded chemical energy at the origins of life. Moving mica sheets may have functioned as enzymes function today, with oscillating motions that push and pull substrate molecules to cause chemical reactions. There are at least three lines of evidence that enzymatic reactions involve protein motion. First, hexokinase is a glycolytic enzyme with no mechanical function. When it binds glucose and ATP, however, a large cleft closes, bringing two domains of the enzyme closer by almost 1 nm [14]. Second, lysozyme motion is greater when an oligosaccharide substrate is present, as compared with lysozyme motion when an inhibitor is present, or when there is no substrate [15]. Third, the mobility of both protein folds and catalytic cycles of enzymes occur on the same timescales [16].

## 3. Membraneless Organelles

Membraneless organelles form by liquid–liquid phase separation under favorable conditions [18,19]. For example, when the protein concentration rises high enough, there is a phase separation of dense protein-containing membraneless organelles from the bulk fluid. Other conditions favor phase separations that produce membraneless organelles. These conditions include changes in pH or salt concentration.

Nucleoli [20] are probably the best known organelles without membranes and are more viscous than water by four orders of magnitude [21]. Nucleoli function in the synthesis of ribosomes in the nuclei of Eukaryotic cells. Membraneless organelles are also found in Prokaryotes [22].

Membraneless organelles are typically composed of RNA and protein and are known as ribonucleoprotein particles (RNPs) [21]. Some of the most ancient RNAs and proteins are found in ribosomes, which are small ribonucleoprotein particles.

The proteins in RNPs typically have Low Sequence Complexity (LSC) and Intrinsically Disordered Regions (IDR) [23]. Prebiotic proteins and peptides are predicted to have low sequence complexity and intrinsically disordered regions, because such proteins and peptides would form more easily than highly structured proteins with complex sequences. Some proteins in RNPs have crossed beta structures; beta structures in proteins are simpler than alpha helices, because beta structures have extended peptide backbones instead of the helical peptide structure of alpha-helices that hydrogen-bond with each fourth amino acid residue in the strand.

Many proteins in RNPs are RNA-binding proteins. Therefore, RNA can nucleate RNP assemblies. Base-paired RNA hairpins partition into membraneless organelles more effectively than unstructured RNA of the same length. Double-stranded nucleic acids are destabilized in RNPs [24,25]. DNA is typically double-stranded, while RNA is typically single-stranded but with intra-molecular base-pairing. RNA appears to be more common at the origins of life than DNA [26]. Many present-day coenzymes are closely related to RNA nucleotides; this is another indication that ribose-containing nucleotides originated early in the emergence of life [27].

Membraneless organelles in living cells maintain their shape and their separation from each other partly because of cellular or nuclear forces acting on them. Crowding and force fluctuations facilitate the formation of these organelles [28]. A small pressure between two coverslips caused membraneless organelles to form in oocytes. Crowding and force fluctuations are also found in the spaces between mica sheets [8]. Force fluctuations occur when fluid flow between mica sheets pushes them apart and together. When the mica sheets are closer together, anything between the sheets will become more crowded.

Sizes of membraneless organelles can scale with the amount of cytoplasm. A single large membraneless organelle is thermodynamically favored over multiple smaller droplets because of the surface tension of the interface. Therefore, membraneless organelles can fuse with time, in a process known as Ostwald ripening [18]. Some RNPs are liquid-phase micro-reactors that speed reactions by concentrating reactants [29]. Molecules diffuse in RNPs and exchange rapidly with the environment.

If RNA and proteins now self-assemble into nucleoli and other organelles without membranes, did they start doing this at life’s origins? Proto-ribosomes might have formed this way. Spaces between mica sheets would have provided a protected environment where this could have happened.

## 4. An Example of Chemical Emergence

The origin of life from non-living materials is an example of chemical emergence. As David Deamer says, “Emergence is now being used in science to connote the process by which a physical or chemical system becomes more complex under the influence of energy… The emergent property is typically unexpected and cannot be predicted” [30].

**Figure 3:**
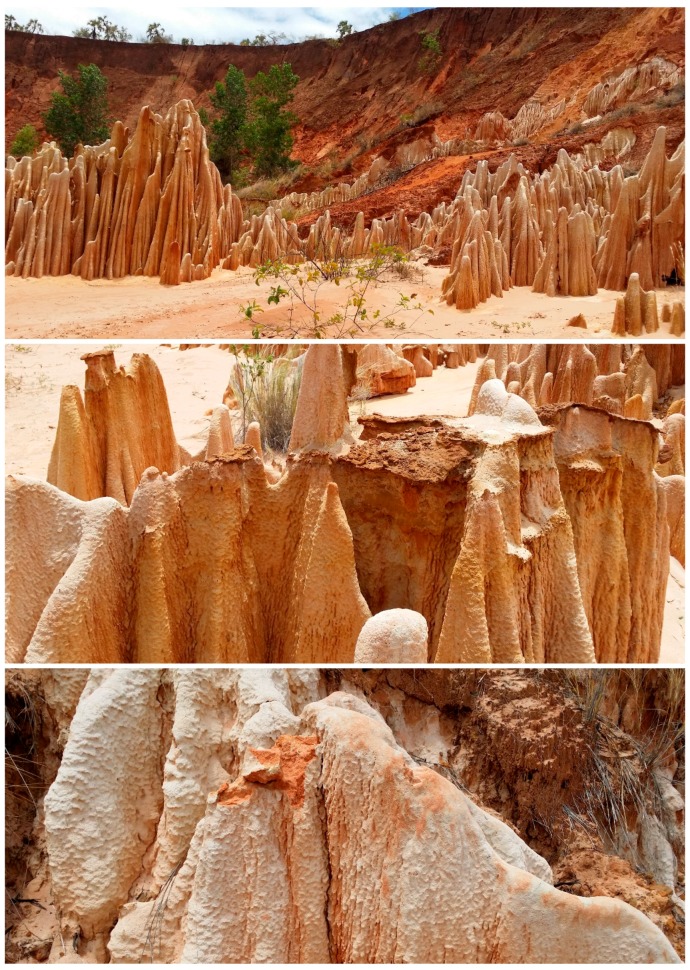
Tsingy Rouge rock formations exposed by erosion in Madagascar are believed to be unique and are an example of chemical emergence by an unknown process. Photos by the author.

Another example of chemical emergence can be found in Madagascar’s Tsingy Rouge (Figure 3). The Tsingy Rouge (Red Tsingy) are unique rock formations found in a single location, near Antisiranana, in northeastern Madagascar [31]. These rock formations have been uncovered by erosion of the surrounding soil. They are composed of laterite, a highly weathered material, rich in iron and/or aluminum, and low in humus [32]. Somehow these unique rock formations formed from matter and energy. The mechanisms of their formation are unknown, at this time. They are thus an analogy for the origins of life. Madagascar’s Tsingy Rouge and the origin of life are both unexpected, and they originated by unknown mechanisms involving matter and energy. The big questions are deceptively simple, for both the Tsingy Rouge and the origins of life: What is the matter? What is the energy?

## 5. Conclusions

Membraneless organelles are found in both Eukaryotes and Prokaryotes today. Their primary components, RNA and protein, are the two essential components of ribosomes, which contain some of the most ancient RNAs [5]. Therefore, membraneless organelles might have been essential for the origins of life. Membraneless organelles have advantages over membrane-bound organelles, because membranes are fragile structures, sensitive to osmotic and other environmental changes. Membraneless organelles may have formed in the spaces between mica sheets, an environment with many advantages for the origin of life. The spaces between mica sheets are a hospitable environment for most of the origins-of-life scenarios, such as the RNA world [26], lipid worlds [33,34], hot or icy origins [35,36], ‘metabolism first’ [37], or even separate origins for replication and metabolism [38].
